# The UV/Visible Radiation Boundary Region (385–405 nm) Damages Skin Cells and Induces “dark” Cyclobutane Pyrimidine Dimers in Human Skin *in vivo*

**DOI:** 10.1038/s41598-018-30738-6

**Published:** 2018-08-24

**Authors:** Karl P. Lawrence, Thierry Douki, Robert P. E. Sarkany, Stephanie Acker, Bernd Herzog, Antony R. Young

**Affiliations:** 1 St. John’s Institute of Dermatology, King’s College London, Guy’s Hospital, London, SE1 9RT UK; 2 University Grenoble Alpes, CEA, CNRS, INAC-SyMMES/CIBEST, 38000 Grenoble, France; 3 BASF Grenzach GmbH, Grenzach-Whylen, 79639 Germany

## Abstract

The adverse effects of terrestrial solar ultraviolet radiation (UVR) (~295–400 nm) on the skin are well documented, especially in the UVB region (~295–320 nm). The effects of very long-wave UVA (>380 nm) and visible radiation (≥400 nm) are much less known. Sunscreens have been beneficial in inhibiting a wide range of photodamage, however most formulations provide very little protection in the long wave UVA region (380–400 nm) and almost none from shortwave visible wavelengths (400–420 nm). We demonstrate photodamage in this region for a number of different endpoints including cell viability, DNA damage (delayed cyclobutane pyrimidine dimers), differential gene expression (for genes associated with inflammation, oxidative stress and photoageing) and induction of oxidizing species *in vitro* in HaCaT keratinocytes and *in vivo* in human volunteers. This work has implications for phototherapy and photoprotection.

## Introduction

Terrestrial solar radiation has long been known to induce damage to the skin. The effects of ultraviolet radiation (UVR) (particularly UVB (280–320 nm) wavelengths) are well established, however the effects of longer wavelength radiation (especially longwave UVA1 (≥380 nm) and visible light (≥400 nm)) are less studied. Visible radiation can penetrate much deeper into the skin than UVB, potentially coming into contact with a greater number of chromophores. It is also a spectral region that causes significant damage in people with photodermatoses such as erythropoietic protoporphyria (EPP) and solar urticaria. EPP is caused by an accumulation of protoporphyrin IX (λ_max_ = 408 nm). UVA1 (340–400 nm) phototherapy is also used to treat a number of skin conditions such as atopic dermatitis, scleroderma and mycosis fungoides. Treatment for these conditions requires exposure to extremely high cumulative doses of UVA1, with some treatment regimes leading to total doses in excess of 2000 J/cm^2^ and individual treatment doses of up to 120 J/cm^2^ ^1,2^. Despite this, photobiology and photoprotection in this region of the solar spectrum are not well studied and poorly understood.

Much of the damage that occurs by the UVA region is attributed to the induction of oxidative stress. This has also been confirmed into the visible region; with one study demonstrating a dose dependent increase in reactive oxygen species (ROS) concentration with doses of visible light equivalent to 20–90 mins of summer sun in Texas, USA^3^. Another *in vivo* study showed that ROS generated by visible light and infrared (IR) radiation account for ~50% of total ROS caused by solar UVR^4^. Production of ROS is significant as they can damage many cellular molecules such as proteins, DNA and lipids^5,6^.

Action spectra have been determined for different types of DNA damage across the UVR and visible regions. UVA1 is much more potent than visible light, but the data are limited with conflicting results^7–9^. The cyclobutane pyrimidine dimer (CPD) is an important lesion in the pathogenesis of skin cancer^10^. Kielbassa *et al*. reported an *in vitro* action spectrum for CPD that shows their generation up to 400 nm^9^. Another study by Liebel showed that CPDs, specifically thymine dimers (T <> T), were not induced by broad-spectrum visible light^3^. This has been further complicated by the recent demonstration of delayed or ‘dark’ CPDs, in melanocytes *in vitro* and pigmented mice by broad-spectrum UVA irradiation, which are formed over 4 hrs post-irradiation, and thought to be due to the chemoexcitation of chromophores such as melanin^11^ however this has also been reported in cells lacking melanin^12^. Melanin has also been shown to play a role in visible light induced DNA damage, resulting in DNA strand breaks, and oxidative pyrimidine modification in melanocytes *in vitro* but not in the melanin deficient (albino) equivalent^13^. The phototoxic potential of melanin and its consequences have also previously been described^11,13–17^.

Both ROS and DNA damage induction have been linked to the expression of a range of genes. Differential expression of mRNA has been demonstrated in human epidermal equivalents exposed to different doses of visible light, with a significant increase in IL-1α, IL-6, GM-CSF and IL-8 mRNA expression^3^. Matrix metalloproteinases (MMP) are thought to be important in skin photoageing^18^. MMP-1 and MMP-9 protein production also increased significantly *in vivo* in response to visible and infrared radiation combined^19^. However, another study found no significant change in MMP-1 protein expression *in vivo* after exposure to blue light^20^. UVR exposure of the skin has also been shown to regulate hormones and the neuroendocrine system, for example in the photoproduction of vitamin D and UV induced stimulation of β-endorphin^21^.

The aim of this study was to investigate the effect of exposure to wavelengths at the UV/visible border region (385–405 nm) using *in vitro* and *in vivo* human models. We investigated a range of endpoints including cell viability, oxidizing species, DNA damage (including delayed CPD) and differential gene expression. This work has consequences for UVA1 phototherapy and photoprotection, as this region is largely neglected by current sunscreen formulations.

## Results

### Cell Viability Reduction *In Vitro*

**Figure 1:**
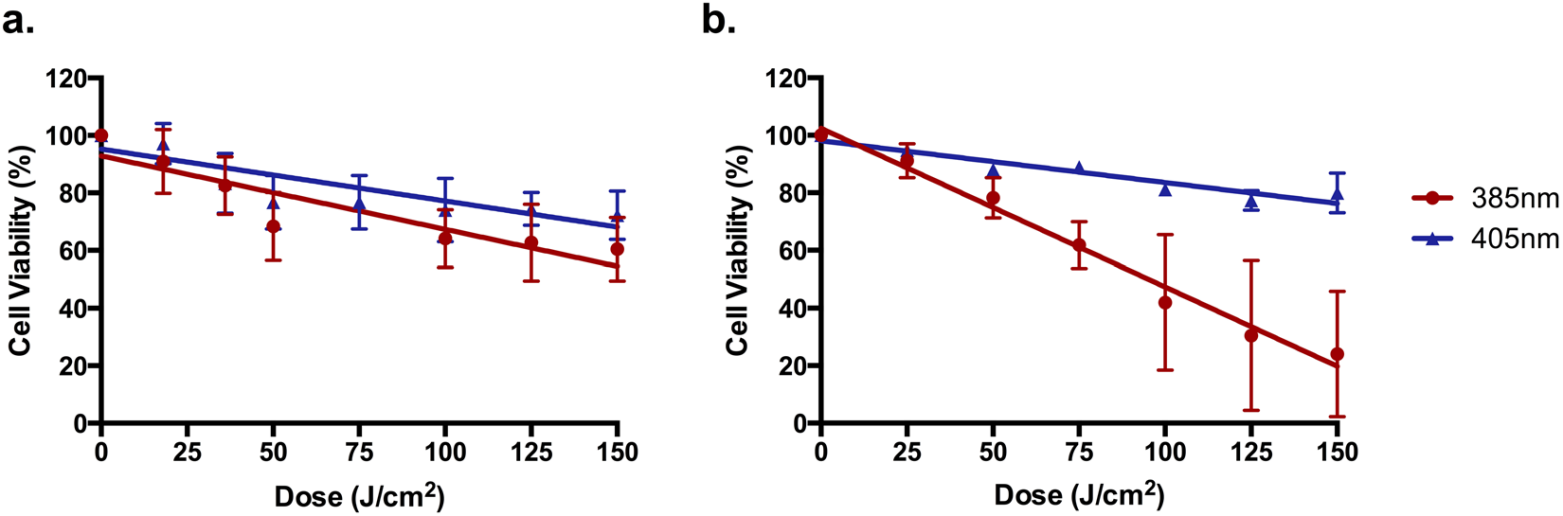
Wavelengths at the UV/visible border (385–405 nm) significantly reduced cell viability *in vitro* in a dose dependent manner. The Neutral Red assay **(a)** showed a significant dose dependent decrease in cell viability for both wavelengths (385 nm-p < 0.0001; 405 nm p < 0.0001; n = 3 linear regression analysis) with no significant difference between the wavelengths (p < 0.1559; n = 3, linear regression analysis). For Alamar Blue **(b)** there was a significant dose dependent decrease in cell viability for both wavelengths (385 nm-p < 0.0001; 405 nm-p < 0.0001; n = 3 linear regression analysis). There was a significant difference between wavelengths (p < 0.0001; n = 3, linear regression analysis). Data points represent the mean ± SD (n = 3).

Cell viability was measured 24 hrs post exposure using the Neutral Red and Alamar Blue methods (Fig. 1). There was a significant dose dependent reduction in cell viability with 385 and 405 nm spectra with both assays. In the case of the Neutral Red assay, there was no significant difference between the wavelengths, however 385 nm was significantly more lethal with the Alamar Blue assay.

### Induction of Oxidizing Species *In Vitro*

**Figure 2:**
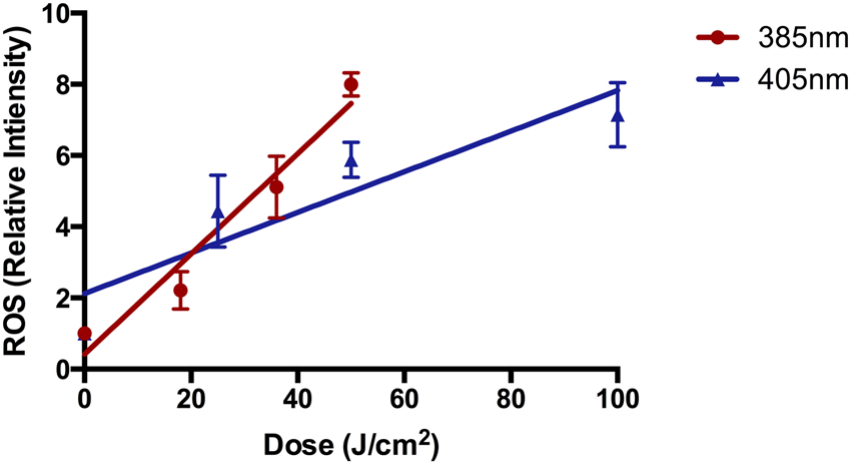
385 nm and 405 nm significantly induced the formation of ROS in a dose dependent manner *in vitro* when measured with the H_2_DCFDA assay. For both sources, there was a significant dose dependent increase in ROS production (385 nm: p < 0.0001, 405 nm: p = 0.0001; n = 3; linear regression analysis) with a significant difference between the slopes of both wavelengths p = 0.0001; n = 3; linear regression analysis). Each point represents the mean ± SD (n = 3).

For both 385 nm and 405 nm there was a significant dose dependent increase in oxidizing species formation (Fig. 2). The slopes of both sources were compared and it was shown that significantly more oxidizing species were produced with 385 nm exposure than 405 nm. The 405 nm source demonstrated good linearity but there was a trend towards a plateau.

### CPD Induction

#### CPD Induction *In vitro*

**Figure 3:**
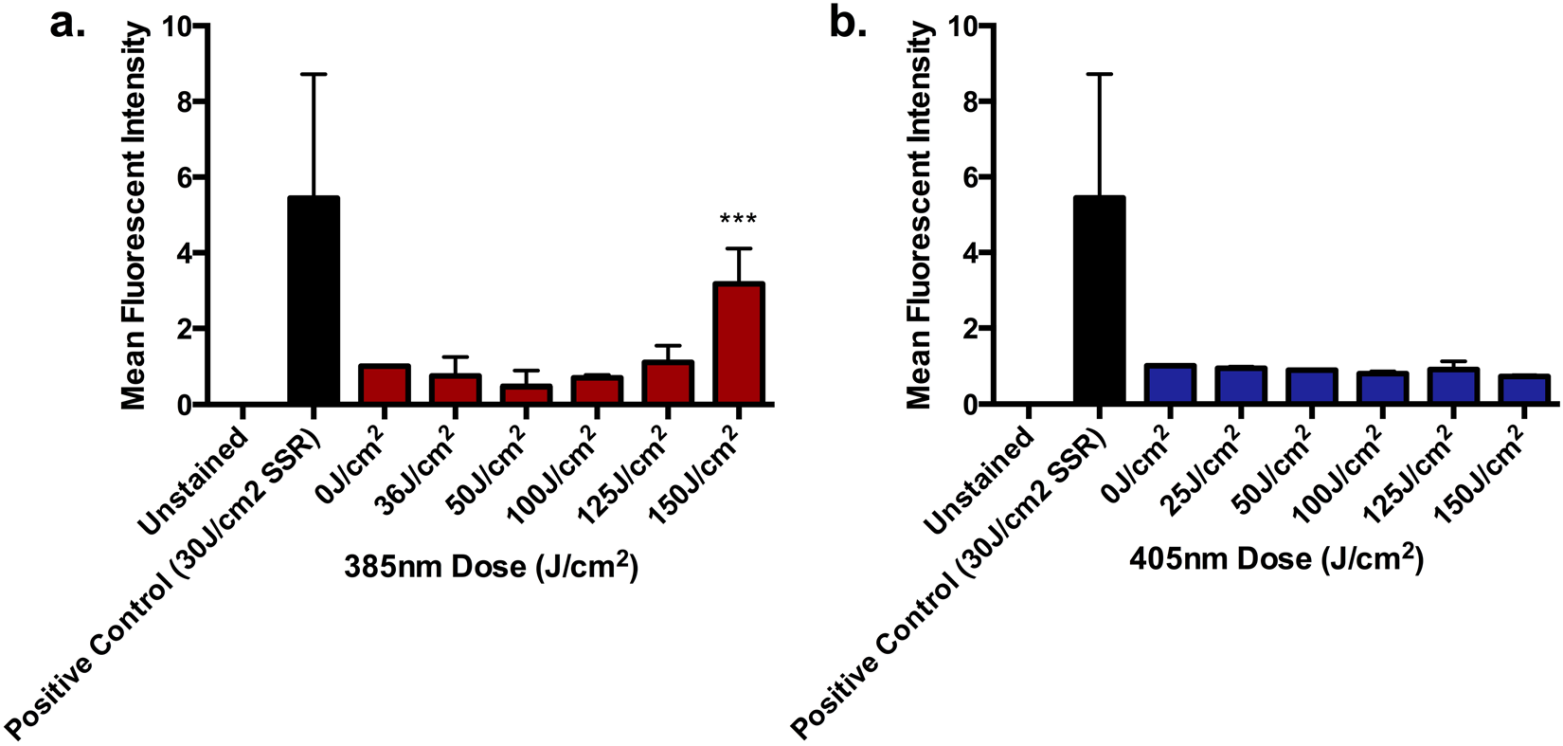
The ability of wavelengths at the UV/visible border (385–405 nm) to induce CPD *in vitr*o when measured by IHC-IF. HaCaT keratinocytes were untreated, or exposed to 0–150 J/cm^2^ of **(a)** 385 nm or **(b)** 405 nm radiation. CPD were measured immediately post exposure using IHC-IF. Columns represent mean ± SD (n = 3). There was a significant increase in CPD at the highest dose of 385 nm radiation (p < 0.0001; n = 3, one-way ANOVA with Dunnett**’**s multiple comparisons test). There was no significant increase in CPD formation at any dose with the 405 nm source.

HaCaT keratinocytes were exposed to acute increasing doses (0–150 J/cm^2^) of each source, and CPD lesions were assessed by IHC-IF immediately post exposure (0 hrs). The 385 nm source produced a significant increase in CPD at 150 J/cm^2^. There was no significant increase detected at any of the doses with the 405 nm source. The results are displayed in Fig. 3.

#### CPD Induction *In vivo* Human Skin

**Figure 4:**
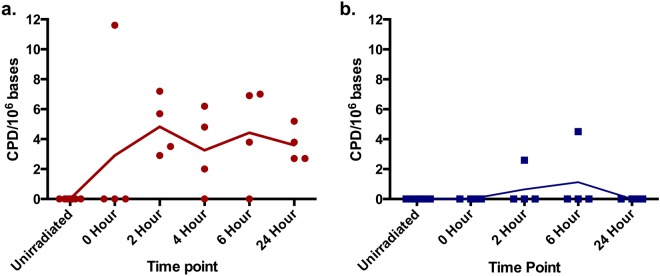
The ability of wavelengths at the UV/visible border (385–405 nm) to induce CPD over time *in vivo* assessed by HPLC-MS/MS. Human volunteers were unexposed, or exposed to 0–150 J/cm^2^ of **(a)** 385 nm or **(b)** 405 nm radiation. Biopsies were taken 0–24 hrs post exposure. DNA was extracted and CPDs were assessed by HPLC MS/MS. Each point represents mean (n = 4 per time point, from 8 volunteers). The high 0 Hour value with the 385 nm source is a probable outlier.

There was an increase in CPD lesions over 2 hrs post exposure with the 385 nm source that was maintained over a 24-hr period with no evidence of repair (Fig. 4). There was a slight increase in CPD with the 405 nm source but this was only detected in two samples. The high value for the 0 hr time point with the 385 nm source is a probable single outlier.

### Gene expression

The effect of the UV/visible border region on differential gene expression was assessed *in vitro* and *in vivo* human skin with responses compared.

#### Gene Expression *in vitro*

**Figure 5:**
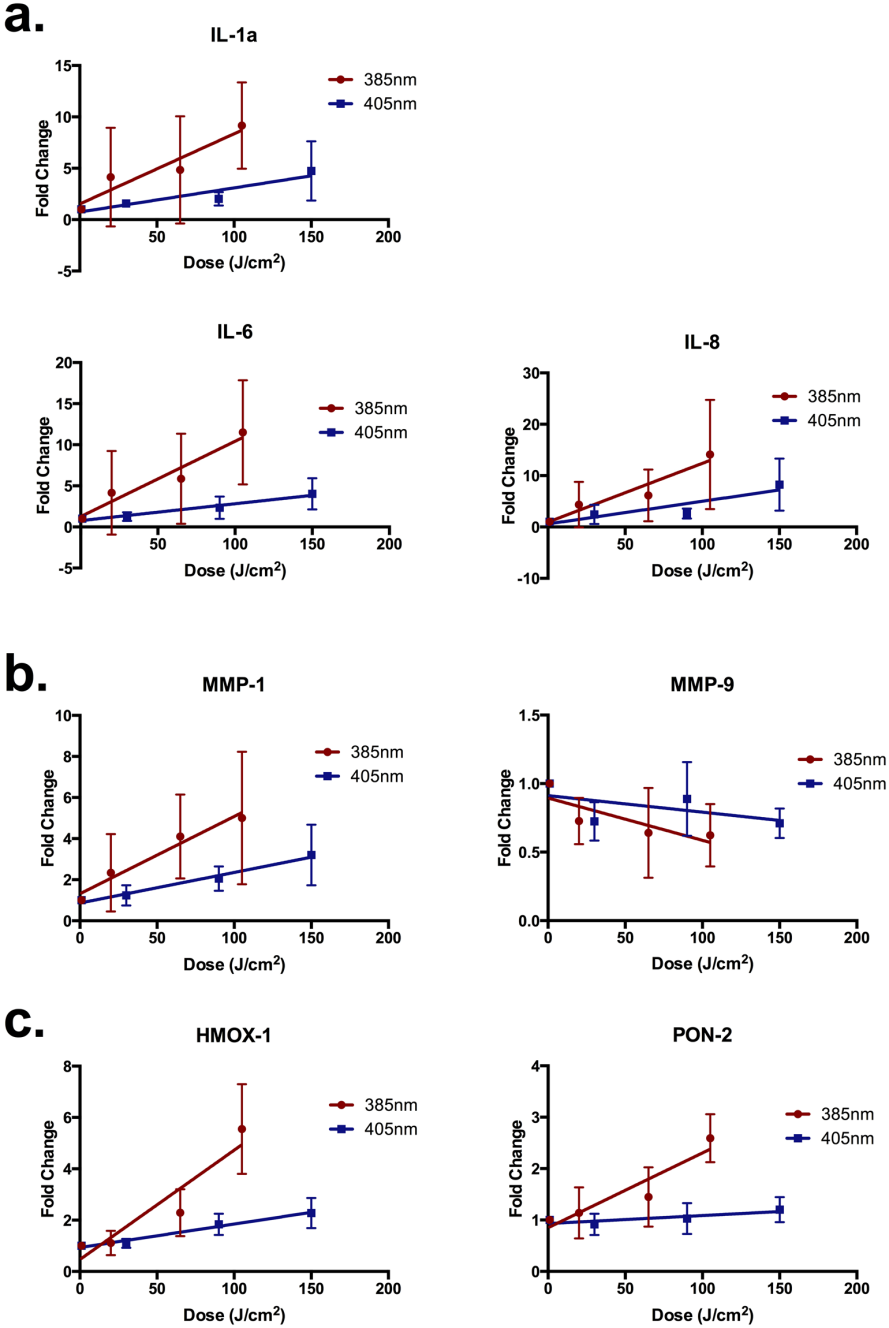
385 nm and 405 nm induced gene expression changes *in vitro*. Genes were categorised based on **(a)** inflammation **(b)** photoageing or **(c)** oxidative stress. HaCaT keratinocytes were exposed to 0–105 J/cm^2^ of 385 nm or 405 nm radiation. Gene expression changes were measured 12 hrs post exposure by qPCR. Columns represent the mean ± SD (n = 4). There was a significant upregulation in many of the genes. The slopes of both 385 nm and 405 nm response were compared and, in general, there was significant fold change in gene expression with all genes with the exception of PON-2, HMOX-1 and IL-6. Results of statistical analysis are displayed in Table 1.

Doses were selected to maintain a cell viability of ≥75%. 12 hrs post exposure, total RNA was extracted from the cells as described in the Materials and Methods. The results are displayed in Fig. 5. The dose response relationship of all the genes was assessed using linear regression analysis and the difference in the slopes between 385 nm and 405 nm compared. The results of the statistical comparisons are displayed in Table 1.

**Table 1:** Results of the statistical analysis of differential gene expression *in vitro*.

Gene	385 nm p value	405 nm p value	385 nm vs. 405 nm
IL-1a	0.0142	**0**.**0027**	0.0596
IL-6	**0**.**0066**	**0**.**0010**	**0**.**0097**
IL-8	**0**.**0082**	**0**.**0030**	0.0580
MMP-1	**0**.**0087**	**0**.**0006**	0.0576
MMP-9	**0**.**0399**	0.1510	0.2298
HMOX-1	**<0**.**0001**	**<0**.**0001**	**<0**.**0001**
PON-2	**0**.**0002**	0.1122	**<0**.**0001**

385 nm was more responsive in all comparisons. Displayed are the linear regression analyses p values (slope) for each gene and wavelength, and a comparison of the slopes between wavelengths. Values in bold represent statistically significant results.

In general there was a significant dose dependent upregulation in gene expression for the majority of genes tested at 385 nm and 405 nm. The 385 nm source was more efficient at inducing an upregulation of gene expression for all tested genes.

#### Gene expression *in vivo*

*In vivo* gene expression was measured by qPCR in skin type I-III human volunteers irradiated with an acute dose of 150 J/cm^2^ of 385 nm and 405 nm, with biopsies taken at 6 and 24 hrs. The details of the volunteers are listed in Materials and Methods. Many genes across all endpoints demonstrated an upregulation in response to both wavelengths. The results for individual genes are displayed in Supplementary Figure S1.

**Figure 6:**
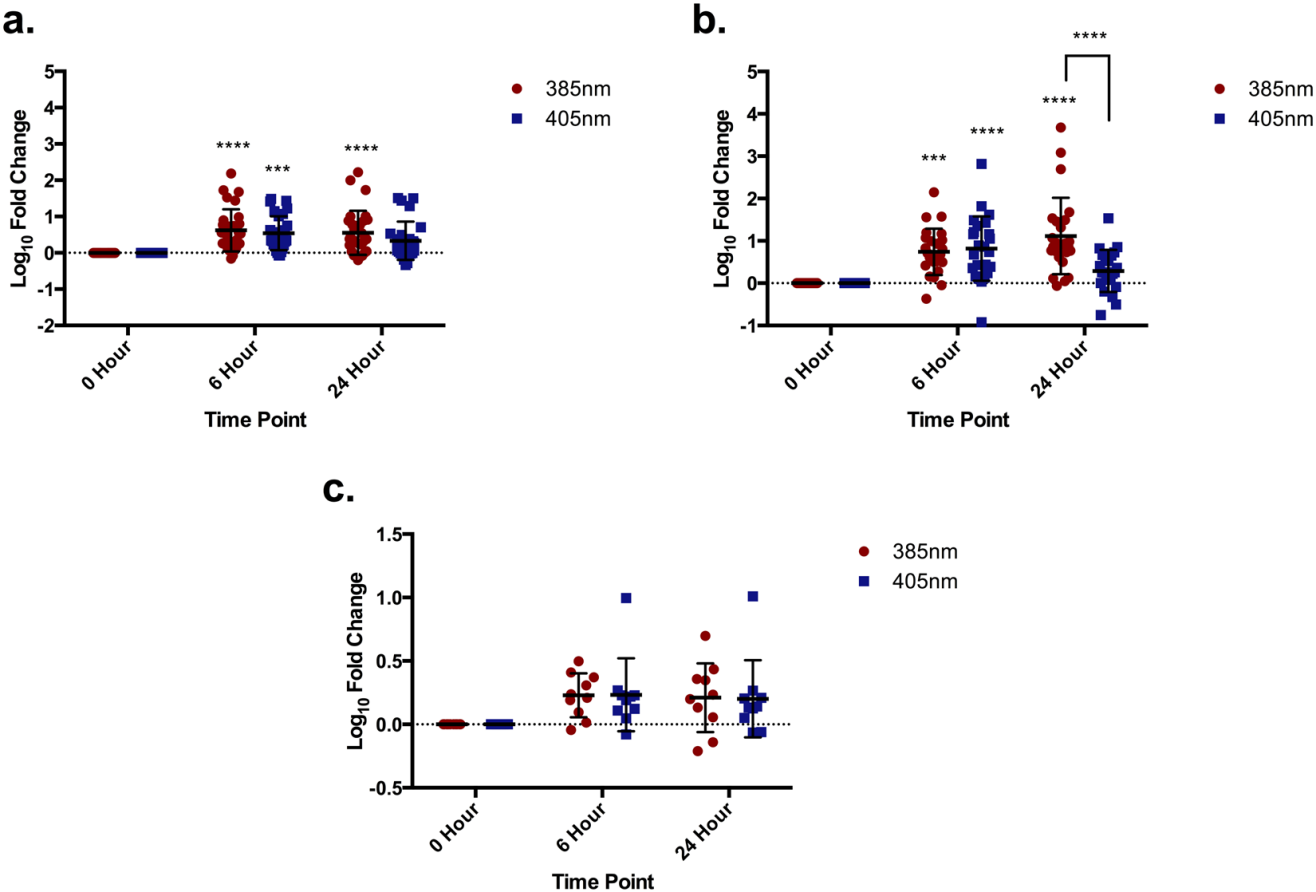
Pooled 385 nm and 405 nm induced gene expression changes *in vivo* at 6 and 24 hrs post exposure. Genes were pooled based on endpoint: **(a)** inflammation, **(b)** photoageing and **(c)** oxidative stress. Volunteers were exposed to 150 J/cm^2^ of 385 or 405 nm radiation. Data are displayed as mean ± SD with the responses of five volunteers pooled based on endpoint. Differences were analysed using two-way ANOVA with Sidak**’**s multiple comparisons test with results displayed in Table 2.

The pooled data are displayed in Fig. 6 and results of the statistical analyses described in Table 2. Genes associated with inflammation and photoageing were significantly upregulated in the treated groups, compared to unirradiated control at both 385 nm and 405 nm. There was no significant increase in genes associated with oxidative stress with either source. In general, there was no significant difference in the upregulation of genes between sources. The one exception was the photoageing genes at 24 hrs, where there was no significant increase at 24 hrs with the 405 nm source. There was however a significant increase at 6 hrs.

**Table 2:** The statistical analysis of pooled gene expression changes.

			Inflammation	Photoageing	Oxidative Stress
ANOVA result	Relationship	Interaction	0.6129	**0**.**0001**	0.9950
		Time	0.2629	**<0**.**0001**	**0**.**0021**
		Wavelength	0.9626	**0**.**0087**	0.9777

A two-way ANOVA with Sidak’s multiple comparisons test was performed on the pooled *in vivo* gene expression data assessing the relationship for time point and wavelengths of light. Values in bold represent statistically significant results.

## Discussion

Terrestrial solar radiation comprises UV, visible and IR radiation wavebands. However, most photobiological studies on human skin have focussed on the UVR spectrum, with many studies concentrating on the shortwave UVB wavelengths. Scientists are beginning to realise the importance of the UVA1, visible and IR parts of the solar spectrum with a recent increase in published studies on these wavebands. One spectral region that is still largely neglected is the UV/visible border (375–415 nm). This region is of particular interest because it is currently poorly protected by many sunscreens and it is used at high doses for UVA1 phototherapy (e.g. lamps with 99.7% UVA1, λ_Max_ = 383 nm). This region is also not currently represented in standard sunscreen testing procedures for both sun protection factor (SPF) and UVA protection factor (UVA-PF), which both use a defined broad-spectrum source that has sharply decreasing output above ~365 nm. The SPF test uses solar simulated radiation (SSR) that is not representative of natural sunlight at wavelengths >365 nm and potentially overestimates the SPF by sunscreens by neglecting this part of the solar spectrum^22^. Exposure to this region is also responsible for the distressing cutaneous symptoms of some photosensitivity disorders such as EPP and solar urticaria, indicating the potential biological activity of this waveband in pathological states. UVA1 and visible light exposure have also been linked to the production of nitric oxide (NO) (linked to blood pressure reduction)^23–25^ and pigmentation^26^ (linked to signalling through skin Opsin3^27^), however these studies focussed on broader and/or different wavebands of radiation from those used in the current study. The sources used in this study cover this neglected interest. Narrowband sources have advantages over a more broad-spectrum source because the relative contribution of each part of the spectrum can be determined. This allows for more robust conclusions pertinent to photoprotection and phototherapy.

The results obtained from Neutral Red and Alamar Blue cell viability assays informed the doses used for later studies. Both assays resulted in a significant dose dependent reduction in cell viability at both wavebands. Neutral Red showed no significant difference between the wavebands, and in both cases, doses as low as 50 J/cm^2^ induced statistically significant death (however doses of over 100 J/cm^2^ were needed to reduce viability below 75%). However, the 385 nm source was significantly more potent than the 405 nm source with the Alamar Blue assay. This may be due to how these assays measure cell viability. Briefly, Neutral Red measures cell membrane integrity whereas Alamar Blue determines reduction potential. Both spectral regions induced oxidative stress, with 385 nm >405 nm. This may lead to Alamar blue measuring a reduced cytoplasmic reduction potential rather than cell viability *per se*.

Generation of ROS by UVA and visible wavelengths is well-established *in vitro*^3,28,29^. The exact cellular photosensitizers remain unknown but β-carotene^30^, porphyrins^31,32^, flavins^29,33,34^ and melanins^11,13–15^ have all been proposed. This damage is particularly important as oxidative stress has been associated with DNA damage, leading to mutations, and protein oxidation, reducing the efficacy of DNA repair enzymes^5,7,35,36^.

The UVR wavelength dependence for most abundant and biologically relevant type of DNA damage, CPD, has been studied, with the non-solar UVC region causing the maximal effect *in vitro*, reflecting the absorption spectrum of DNA^37^. In practice, most CPD in the skin are caused by solar UVB^38^, but there are numerous reports of UVA-induced CPD, although with a lower efficiency, and many of these studies focus on the shorter end of the UVA region (<370 nm)^9,39–43^.

There are conflicting reports about CPD production by the UV/visible boundary region^3,7^. No CPD were detected *in vitro* immediately post exposure with the highest 405 nm dose (150 J/cm^2^) but the same dose of 385 nm resulted in a low but significant increase, with none detected at lower doses. This supports reports of a slight increase in CPD with UVA1 in AS52 Chinese hamster cells but virtually none with visible radiation (doses up to 150 kJ/m^2^)^9^. The 385 nm *in vivo* data provide the first evidence for ‘dark’ CPD in human skin *in vivo* as they increased over 2 hrs and persisted for 24 hrs. These results suggest the formation CPD by a route other than direct absorption of radiation by DNA as no CPD were detected immediately post irradiation (ignoring the single outlier)^11,12^. Melanin has also been shown to play a role in DNA damage caused by visible light, with strand breaks, FPG and EndoIII sensitive lesions detected^13^. Despite melanin acting as an excellent photoprotectant^44^ (particularly in darker skin types), there is evidence for its photosensitizing potential through the photogeneration of ROS^45–47^. Other sensitizers may also act in a similar way^48^ and multi-chromophore excitation may be involved. Melanin photosensitization could explain the results of Tewari *et al*., who reported increased UVA1-induced CPD with epidermal depth in human skin *in vivo*, (in direct contrast with the results observed with UVB (300 nm)) given that melanin concentration also increases with epidermal depth^43,44^. The quantitative CPD assay required the destruction of the tissue so we lack information on the localization of the lesions. It could be expected that other endogenous skin chromophores might cause CPDs through similar mechanisms^12^. Very unusually, there was no evidence of repair even at 24 hrs post exposure, at which time there is usually good repair with CPD induced by SSR^49^. There are a number of possible explanations for this. Firstly the levels of CPD are relatively low, so perhaps there is a threshold that must be reached before the DNA damage repair response is triggered. Another possible explanation is that there is so much oxidative stress induced that it damages the DNA repair enzymes and leads to a loss of function (as previously demonstrated by broadband UVA irradiation^5,50^) or there is a longer-term production of CPD (through photo/chemo-excitation mechanisms^11,12^) that masks any repair. This lack of repair suggests that there will be an accumulation of CPD with repeated lower level exposure, as has been demonstrated with repeated sub-erythemal exposure with SSR^51^. Here it should be noted that the exposures given in the current manuscript were sub-erythemal (manuscript in preparation). The generation of ‘dark’ CPD by longer wavelength UV/light, may have clinical significance for skin cancer even if the number of CPD is relatively low compared to UVB. The spectral dependence of “dark” CPD is unknown and its elucidation may help with the identification of chromophores. Skin cancers result from mutations in keratinocyte stem cells and melanocytes, which reside in the basal epidermis at the epidermo-dermal junction. The deeper penetration of these longer wavelengths means that CPD created by longer wavelength UVA and visible light are more likely to occur in the cells responsible for skin cancer induction. Thus, the carcinogenic potential of these longer wavelengths may be greater.

UVR has been widely implicated in differential gene and protein expression, with different spectral regions responsible for different effects. The endpoints selected were based on our previous human *in vivo* work^52^. These comprised three main categories: inflammation/immunoregulation, matrix metalloproteinases (as a marker of photoageing) and oxidative stress. Changes in gene expression were initially measured *in vitro* and then in *vivo*. The *in vitro* results demonstrate significant increases in many of the genes for all categories for both 385 nm and 405 nm. The exceptions were MMP-9 and PON-2. MMP-9 expression was slightly down regulated with 385 nm with no effect at 405 nm. For PON-2 there was a significant upregulation with 385 nm that was not seen with 405 nm. The difference in PON2 expression can be explained by the greater oxidative potency of 385 nm compared to 405 nm, however the difference with MMP-9 needs to be investigated further. Neither PON-2 nor MMP-9 showed any effect at 405 nm. These differences may reflect different chromophores for different genes. It is evident that both wavelengths significantly induce markers of inflammation and photoageing at a cellular level, with the upregulation of HMOX-1 corroborating with the data from the oxidizing species assay.

The *in vivo* human data demonstrate a much greater increase and range of responses in gene expression changes compared to the *in vitro* data, however the general trends are comparable and similar to UVR responses reported by previously^52,53^. Combining the *in vitro* DNA damage and photoageing results is particularly interesting as there is some debate about the trigger for the expression of MMPs. Some studies suggest it is the formation of CPDs whilst others link this to oxidative stress, but this may vary with different MMP^19,53–55^. The doses used in the gene expression studies induced no/few measurable CPDs with 405 nm but significantly increased MMP mRNA; suggesting independence from CPD, but related to oxidative damage. The different time courses of MMP expression observed in *vivo* with UVB/broadband UVA or the visible border region may be due to MMP expressed via one of these two possible mechanisms^3,53,54^. There was no significant change in genes linked to oxidative stress (despite the increase in ROS detected *in vitro*), matching the previous studies by Tewari *et al*.^52^, but the *in vitro* gene expression experiments showed more significant changes. This suggests better anti-oxidant defence mechanisms *in vivo*.

Gene expression responses *in vitro* and *in vivo* were compared to justify the use of the *in vitro* model (Supplementary Figure S2), as there are concerns with the use of HaCaT keratinocytes with a p53 mutation^56^ (a protein important in cell cycle and repair). In general, there was no significant difference in the magnitude of the responses of the genes after irradiation with either 385 nm or 405 nm between *in vitro* and *in vivo* experiments. The only exception was with MMP-1 induction with both 385 nm and 405 nm, where MMP-1 was significantly more upregulated *in vivo* compared to *in vitro*, although the trends were same. These results validate the use of the *in vitro* HaCaT keratinocyte model for gene expression studies, as overall there is an excellent correlation between the *in vitro* model and the *in vivo* response. A significant benefit of this work is the same sources and experimental conditions were shared between *in vitro* and *in vivo* studies – this is not the case in many comparisons.

The highest doses used in this study are slightly higher than would be possible to receive in a full day’s exposure to the sun in a tropical environment, although many of the effects were observed at lower doses that would be possible^57^. The doses are particularly relevant to UVA1 phototherapy that uses high dose (>60 J/cm^2^) exposure to a source peaking in the UVA1 waveband at 383 nm several times a week (3–5 sessions) for many weeks (typically 10 weeks). This can lead to extremely high cumulative doses and there is little known about the long-term safety of this treatment^58^. This study only used a single exposure and found significant damage, so the effect of repeated exposures needs to be investigated.

In conclusion, the data demonstrate that the UV/visible boundary wavelengths cause significant biologically relevant damage *in vitro* and *in vivo*, including dark CPD formation. This damage is most likely caused by oxidative stress generated by chromophores in the skin such as protoporphyrin IX, β-carotene and melanins that absorb strongly in this region, although this would require further investigation. These effects have implications for the possible need to extend photoprotection in the normal population to longer wavelengths, and for UVA1 phototherapy. They raise the need for photoprotection in this region and the inclusion of wavelengths longer than 380 nm in sunscreen testing protocols.

## Materials and Methods

### Radiation sources

**Figure 7:**
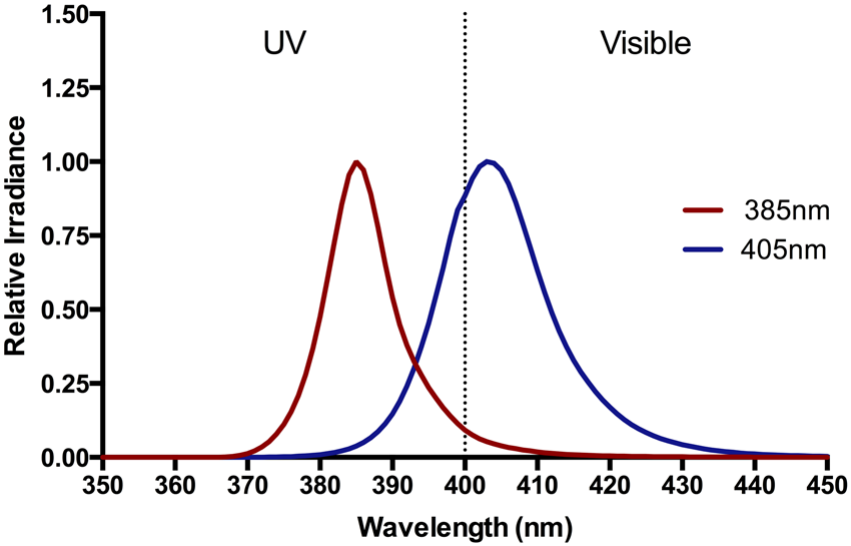
The spectral outputs of the experimental sources. The spectral output of the 385 nm and 405 nm Loctite LED sources used in all the studies. The full width at half maximum (FWHM) of the sources were 7 nm and 10 nm respectively. Spectra (350–450 nm) were measured with a Bentham spectroradiometer.

The radiation sources used were Loctite LED flood array systems with peak outputs at 385 and 405 nm (Loctite, Henkel Ltd, UK). Each array has an irradiation surface of 97 mm × 96 mm consisting of 144 LED. The spectral irradiances of both sources are shown in Fig. 7. Spectra were measured using a DM120BC double-monochromator spectroradiometer (Bentham Instruments, Reading, UK) using an integration sphere, calibrated by the Centre for Radiation, Chemical and Environmental Hazards (CRCE), Public Health England (PHE) against a UK national standard. Irradiance of the sources was routinely measured with a Loctite UVA/Vis radiometer (Loctite, Henkel Ltd, UK). Typical irradiances were 74 mW/cm^2^ for the 385 nm source and 260 mW/cm^2^ for the 405 nm sources, with irradiation times for the highest dose of 150 J/cm^2^ of 34 minutes and 9.5 minutes respectively. Solar simulated radiation (SSR) was used as a positive control in some assays and the source used has been described previously^59^.

### Cell culture

The immortalised human keratinocyte cell line HaCaT was purchased from ATCC (Manassas, Virginia, USA) and cultured in 48 well plates in Dulbecco’s modified Eagle’s medium (DMEM; Invitrogen, Paisley, UK), supplemented with 10% (v/v) foetal calf serum (FCS), 100 U/ml penicillin and 100 μg/ml streptomycin (Invitrogen Paisley, UK). Cells were incubated in a humidified atmosphere (5% CO_2_ at 37 °C).

### Ethical approval & volunteer recruitment

The *in vivo* study was approved by the National Research Ethics Service City and East (Ref: 13/LO/0380) and the Guy’s and St Thomas’ NHS Foundation Trust Research and Development department (Ref: RJ115/N205) and conducted in accordance with the Declaration of Helsinki Principles. All volunteers gave written informed consent. Volunteers of skin types I-III were recruited with details described in Table 3.

**Table 3:** Details of human volunteers and the endpoint for which samples were used.

No.	Age	Sex	Skin Type	Endpoint
1	21	M	II	Gene Expression
2	32	F	II	DNA Damage + Gene Expression
3	26	M	III	DNA Damage + Gene Expression
4	27	M	II	DNA Damage + Gene Expression
5	21	F	III	DNA Damage + Gene Expression
6	28	F	I	DNA Damage
7	23	M	II	DNA Damage
8	27	M	II	DNA Damage
9	18	M	II	DNA Damage

### Biopsy procedure

Punch biopsies (4 mm diameter) were taken under local anaesthetic from the centre of each treated site at different time points (0–24 hrs) post exposure. An adjacent non-exposed site was also biopsied. Biopsies were divided into two halves. Biopsies for DNA damage analysis were immediately snap frozen in liquid nitrogen and stored at −80 °C until transport on dry ice to Grenoble, France where they were stored at −80 °C until analysis. Biopsies for gene expression were placed in RNA later and stored at −80 °C until extraction.

### Irradiation procedure

#### *In vitro*

All assays were performed on HaCat cells grown as a 70–80% confluent monolayer in multiwell plates. Cells were were washed three times in phosphate buffered saline (PBS) (Invitrogen, Paisley, UK). The cells were then irradiated with either 385 nm or 405 nm radiation (0–150 J/cm^2^) as a single expsoure. Doses were selected based on those that are environmentally relevant and used for phototherapy, as well as refelecting the literature. Temperature was controlled with a cooling platform. After irradiation, PBS was removed and replaced with media or processed immediately depending on the experimental design.

#### *In vivo*

1 cm^2^ irradiation sites on the upper sun-protected buttocks of healthy volunteers were exposed to a dose of 150 J/cm^2^ of 385 nm or 405 nm light (as a single exposure) from a distance of 15 cm from the source. An adjacent site was left unexposed for the control biopsy. A fan was used to cool irradiation sites to ensure the skin did not overheat.

### Cell Viability

#### Neutral red assay

Cell viability was measured 24 hrs post-irradiation using the Neutral Red uptake assay. Neutral Red solution (4 μg/ml in growth medium) (Sigma, Gillingham, UK) was added to the cells and incubated at 37 °C, 5% CO_2_ for 2 hrs. Cells were washed three times in PBS and the destain solution (50% v/v ethanol, 49% v/v ddH_2_O, 1% v/v glacial acetic acid) was added. Optical density was measured at 540 nm using a Spectra Max 384 Plus spectrophotometer (Molecular Devices, Sunnyvale, California, USA).

#### Alamar blue assay

Alamar Blue solution (1/10^th^ of total growth medium volume) (ThermoFisher, Waltham, Massachusetts, USA) was added to the cells  which were incubated at 37 °C, 5% CO_2_ for 4 hrs and protected from light. The excitation/emission = 540–570/580–610 nm was used to measure fluorescence using a Spectra Max 384 Plus spectrophotometer. Each condition was tested in triplicate and the average calculated.

### Measurement of oxidizing species

Levels of ROS were assessed using the probe carboxy-H_2_DCFDA. It is however accepted that other species may also oxidize this probe to its fluorescent product and this must be considered and hence the term oxidizing species is used^60^. Cells were treated and immediately incubated with 10 μM carboxy-H_2_DCFDA (Invitrogen, Paisley, UK) in PBS for 30 mins in the dark at 37 °C, 5% CO_2_. Cells were washed in PBS, trypsinised for 10 mins at 37 °C, centrifuged at 1200 rpm for 5 mins at room temperature and then resuspended in PBS and counterstained with 4’,6-diamidino-2-phenylindole (DAPI) for analysis by fluorescence activated cell sorting (FACS) using a Becton Dickinson FACSAria II (Becton Dickinson, Franklin Lakes, New Jersey, USA). Cells were gated to only analyse live cells (DAPI negative) and the average mean green intensity per condition was then plotted from at least 10,000 measured events. Analysis was carried out using FlowJo 8.7 software (Flowjo LLC, Ashland, Oregon, USA).

### Immunocytochemistry – imunnofluorscence (IHC-IF) for CPD

Cells were treated and immediately washed, then fixed in 2% (v/v) paraformaldehyde with 0.5% (v/v) Triton X-100 in PBS for 30 mins at 4 °C. DNA was then denatured by incubation in 2 M HCl for 10 mins at 37 °C. Non-specific sites were blocked using blocking buffer of 20% (v/v) goat serum and 0.1% (v/v) triton x-100 in PBS for 30 mins at room temperature. Anti-CPD antibody (Clone TDM-2 which recognizes TT, TC, CT and CC CPD lesions) (Cosmobio, Tokyo, Japan) was added at 1:1000 in blocking buffer for 1 hr at room temperature. Alexa Fluor® 488 was diluted in blocking buffer (1:200 dilution) and incubated for 1 hr at room temperature and finally DAPI was added for 10 mins. Washing was carried out with PBS (3 × 5 mins) between each step. Image capture of cells was carried out using a Ziess Axio-Observer Z1 Microscope (Carl Zeiss, Cambridge,U.K.) with AxioVision V.4.8 software (Carl Zeiss). Image analysis was carried out using Cell Profiler v.2.1.1 (Broad Institute, Cambridge, MA, U.S.A.), gating around the nucleus of each cell and the relative mean green intensity (CPD staining) of each nucleus was measured (~150 nuclei measured per condition). The mean of the nine pictures was determined and used as the end point.

### HPLC tandem mass spectrometry (HPLC-MS/MS)

DNA damage *in vivo* was assessed at 0, 2, 4, 6 and 24 hrs post-exposure by HPLC-MS/MS as previously described^61^. Results were expressed as the number of bipyrimidine photoproducts/10^6^ normal bases. The analysis was for thymine dimers (TT) because they are the most frequent CPD. HPLC-MS/MS was only used for *in vivo* samples, as it is the most sensitive method available and gave the greatest chance of detecting lesions in the best available model.

### RNA extraction and qRT-PCR

RNA was extracted using the mirVana miRNA Isolation kit (Life Technologies, Paisley, UK) RNA was reversely transcribed to cDNA using the High Capacity cDNA Reverse Transcription Kit (Applied Biosystems, Paisley, UK) as previously described^59^. qPCR was performed using TaqMan Gene Expression Assays (Applied Biosystems, Massachusetts, USA) according to the manufacturer’s protocols. GAPDH was used as the housekeeping gene. Gene fold change was measured using the ΔΔCT method^62^. Gene selection was based on previous *in vivo* human studies^52^ and the literature.

For *in vivo* data, many genes showed a large fold change, but when tested statistically this was not significantly different from the non-irradiated controls The normality of the pooled data was tested and it was found that data were not normally distributed when assessed by the D’Agostino and Pearson test (p < 0.0001) so the data were logged to normalise the data before further analysis^63^. Genes with similar end points were pooled (for inflammation, photoageing and oxidative stress), and two-way ANOVA was performed. This approach led to more robust data.

### Statistical analysis

All data are expressed as the mean ± standard deviation (SD) where n ≥3. Statistical analyses were carried out using Graphpad Prism 6.0 (Graphpad Software, San Diego, CA) and were evaluated using the student’s t-test, ANOVA, linear and non-linear regression. Significance limits were set as: *=p ≤ 0.05, **=p ≤ 0.001, ***=p ≤ 0.0001.

## Electronic supplementary material

Supplementary Materials

## Data Availability

All data generated or analysed during this study are included in this published article (and its Supplementary Information files).
