# Supplementary material for: The UV/Visible Radiation Boundary Region (385–405 nm) Damages Skin Cells and Induces “dark” Cyclobutane Pyrimidine Dimers in Human Skin in vivo
Source: Sci Rep. 2018 Aug 24;8:12722. doi: 10.1038/s41598-018-30738-6 (PMC6109054; doi:10.1038/s41598-018-30738-6)
Supplement: Supplementary file 1 — Supplementary Materials [file 41598_2018_30738_MOESM1_ESM.docx]

The UV/Visible Radiation Boundary Region (385 – 405 nm) Damages Skin Cells and Induces “dark” Cyclobutane Pyrimidine Dimers in Human Skin *in vivo*

Karl P. Lawrence^1*^, Thierry Douki^2^, Robert PE. Sarkany^1^, Stephanie Acker^3^, Bernd Herzog^3^, Antony R. Young^1*^

^1^ St John’s Institute of Dermatology, King’s College London, London, UK.

^2^Université Grenoble Alpes, SyMMES, & CEA, INAC, SyMMES, LAN, F-38000 Grenoble, France

^3^BASF Grenzach GmbH, Grenzach-Whylen, Germany

***Correspondence to** [**karl.lawrence@kcl.ac.uk**](mailto:karl.lawrence@kcl.ac.uk) **or** [**antony.young@kcl.ac.uk**](mailto:antony.young@kcl.ac.uk)

**SUPPLEMENTARY MATERIALS**

## S1: Individual *in vivo* gene expression changes

Figure S1: 385 nm and 405 nm induced gene changes *in vivo*. Volunteers were exposed to 0 or 150J/cm^2^ of 385 or 405 nm radiation. Biopsies were taken 0, 6 or 24 hrs post exposure and gene expression changes were measured by qPCR and categorised based on (a) inflammation (b) photoageing and (c) oxidative stress. Each point represents the mean ± SD (n=5).

## **S2: *In Vitro* – *In Vivo* Gene Expression Comparisons**

The *in vitro* and *in vivo* gene expression responses were compared (Figure S2) to assess the validity of the *in vitro* model. The time points used for studies were different so direct time comparison was not possible but general responses were compared for genes that were measured using both methods.

Figure S2. Comparison of *in vitro* and *in vivo* 385 nm and 405 nm induced gene changes. The *in vitro* and *in vivo* responses to 385 nm and 405 nm irradiation were compared to assess the validity of *in vitro* studies. Differences were analysed using two-way ANOVA with Sidak’s multiple comparisons test, comparing the fold change differences between *in vitro* response at 12 hrs with the *in vivo* response at 6 and 24 hrs. There was no significant difference between methods used with the exception of MMP-1, which showed the same trend, but a different magnitude of response (385 nm: p=0.0.0915, 405 nm: p=0.6083). The response at 6 and 24 hrs *in vivo* were also compared with the same outcome; in general there was no significant difference with the exception of MMP-1. Columns represent the mean ± SD (*in vitro* n=4; *in vivo* n= 5)
